# The Effect of an Anthropogenic Magnetic Field on the Early Developmental Stages of Fishes—A Review

**DOI:** 10.3390/ijms22031210

**Published:** 2021-01-26

**Authors:** Krzysztof Formicki, Agata Korzelecka-Orkisz, Adam Tański

**Affiliations:** Department of Hydrobiology, Ichthyology and Biotechnology of Reproduction, West Pomeranian University of Technology in Szczecin, K. Królewicza 4, 71-550 Szczecin, Poland; akorzelecka@zut.edu.pl (A.K.-O.); adam.tanski@zut.edu.pl (A.T.)

**Keywords:** anthropogenic magnetic field, fish, early developmental stages, embryogenesis, larval development, gametes

## Abstract

The number of sources of anthropogenic magnetic and electromagnetic fields generated by various underwater facilities, industrial equipment, and transferring devices in aquatic environment is increasing. These have an effect on an array of fish life processes, but especially the early developmental stages. The magnitude of these effects depends on field strength and time of exposure and is species-specific. We review studies on the effect of magnetic fields on the course of embryogenesis, with special reference to survival, the size of the embryos, embryonic motor function, changes in pigment cells, respiration hatching, and directional reactions. We also describe the effect of magnetic fields on sperm motility and egg activation. Magnetic fields can exert positive effects, as in the case of the considerable extension of sperm capability of activation, or have a negative influence in the form of a disturbance in heart rate or developmental instability in inner ear organs.

## 1. Introduction

The number of sources of anthropogenic magnetic fields (MFs) and electromagnetic fields (EMFs) that affect fishes increases constantly with technological advancement. This is due to the increasing number of electricity-generating and transferring devices in water: electric power stations, wind farms, pipelines with a cathodic protection system, and, most of all, high-power transmission cables [1,2,3,4,5,6,7]. The industrial development with its increasing demand for electricity has caused the development of transmission lines, both over ground and underwater, which implies progressing, significant changes in aquatic environments [8].

Overall, underwater cables can be assigned to two categories. The first includes high voltage direct current cables (HVDC), which emit static magnetic fields (SMFs); the second includes alternating current cables (AC), which emit time-varying EMFs [4]. The characteristics of these two categories are different, and, in consequence, they have different effects on fishes. It is assumed that low-frequency electromagnetic fields (LF-EMFs) exert a much stronger effect on biological structures than do SMFs [8,9]. MFs, to which fishes may become exposed for rather short distances from the source, and the field’s magnitude depend on the type of the cable, on the electric current intensity, and on whether the cable rests on the sea bottom or is embedded in it [2,4]. The intensity of the field over these cables increases in rough proportion to the current flow in the cables and depends on the depth at which the cable is buried [2]. DC values of MFs recorded in close proximity of underwater cables can reach <10 mT [9], and those near industrial cables at the level of sea bottom can reach 250 µT [2,3] and can be perceptible by fishes within a distance of several meters [10].

We review the effect of MF and EMFs on early developmental stages, with special reference to gametes, embryogenesis (its duration and changes during the process), the survival of the embryos, changes in larval behavior, and pigmentation; during these stages, when cell differentiation takes place, the developing organism is most susceptible to the effects of such factors, and the consequences of these effects extend to the whole life span of the fish and even to future generations.

## 2. Embryogenesis

Fish embryogenesis is sufficiently long-lasting for its course to become modified when the developing embryo is placed in a distorted geomagnetic field (GMF); [11,12] the modifications depend on the value and characteristics of the static or time-varying field of anthropogenic origin [13,14,15].

### 2.1. Duration of Embryogenesis and Hatching

Artificially generated SMFs (from 1 to 5 mT) have an effect on the duration of embryonic development of the sea trout (*Salmo trutta* m. *trutta* L.) and rainbow trout (*Oncorhychus mykis* (Walbaum)). When applied to developing embryos during the whole incubation period from the fertilization to the hatching of eleutherembryos, such fields slow down embryonic development and, thus, extend the time spent by the embryos inside the egg envelopes [11]. SMFs of 1 mT, 3 mT, and 5 mT were observed to slow down the embryonic development of the sea trout: in control conditions, the embryogenesis took 245 D° (sum of mean daily temperature of each day of incubation counted from fertilisation to hatching), and the values were 260 D°, 268 D°, and 288 D° in SMFs of 1 mT, 3 mT, and 5 mT, respectively [16]. A similar slowing down of embryonic development was observed in European whitefish (*Coregonus lavaretus* (L.)) (control conditions: 399 D°; in MFs of 1 mT and 3 mT: 405 D°; in 5 mT: 414 D°) as well as in the vendace (*Coregonus albula* (L.)) (control: 331 D°; in MFs of 1 mT, 3 mT, and 5 mT: 373 D°, 382 D°, and 397 D°, respectively) [16].

Exposure to an SMF of 10 mT during embryogenesis of the northern pike (*Esox lucius* L.) resulted in a shortening of the embryogenesis by 1 day compared to the control [17]. The authors attributed this effect to a change in metabolic rate; changes in the heart rate of embryos and larvae of the northern pike in fields of 4 mT [18] and 50–70 mT were interpreted in a similar way [11]. Incubating rainbow trout eggs in an STM (10 mT) and an EMF (1 mT, 50 Hz) from the eyed stage to hatching caused no significant changes in the duration of embryogenesis [12]. The exposure of zebrafish (*Danio rerio* (Hamilton)) embryos 48 h post-fertilization to a 50 Hz AC MF of 1000 µT resulted in a delay of hatching compared to the control. The moment of exposure to MF was crucial, since no statistically significant effect was observed when the exposure started 2 h post-fertilization [19]. A sinusoidal EMF (150 µT, 500 Hz), applied to developing roach (*Rutilus rutilus* (L.)) embryos from the moment of fertilization, shortened the duration of embryogenesis, and the embryos hatched earlier compared to the control [20]. The hatching of zebrafish embryos continuously exposed to 50 Hz sinusoidal fields of 200 µT, 400 µT, and 800 µT 48 h post-fertilization was delayed compared to the control [21].

The successful leaving of egg envelopes is crucial to larval survival. The secretion of a hatching enzyme (chorionase) to the perivitelline space of the egg, causing the egg envelope degradation and, consequently, the hatching of the embryo, may depend on an MF [22,23]. An SMF of 10 mT caused no statistically significant difference between the experimental variant and the control in the hatching success of the northern pike. The percentage of hatching embryos was very similar: 87.1% in the experimental variant and 83.3% in the control [17]. A shortening of the hatching period in SMFs of 4 mT and 5 mT compared to the control was also observed: the duration of the hatching (time between leaving the egg envelopes by the first and the last embryos) of sea trout larvae was 4 days in an MF and 15 days in the control [11]. Similarly, a sinusoidal EMF (150 µT, 500 Hz), applied to developing roach embryos from the moment of fertilization, resulted in a longer hatching process compared to the control [20].

### 2.2. Survival and Size of Eleutherembryos

MF and EMF effects on the duration of embryogenesis, hatching, and survival are species-specific; they depend not only on the intensity of the field, but especially on the moment of exposure during embryogenesis.

The value of a generated MF can affect the characteristics of hatched sea trout eleutherembryos. An SMF of 5 mT was observed to cause changes in the mass and length of eleutherembryos: the eleutherembryos, since fertilization had been incubated in the MF of this intensity, were longer, heavier, and more mobile. The difference in length was up to 3.79 mm (14.8%), and the difference in body mass up to 31.33 mg (26.5%) [11]. In the rainbow trout, the difference between embryos incubated in a generated SMF of 5.5 mT and those in control conditions was small—the mean length of the larvae from eggs incubated in the SMF was 15.25 mm, and that in the control was 12.77 mm, while the respective values for body mass were 78.3 mg and 61.3 mg [11]. In contrast, a sinusoidal EMF (150 µT, 500 Hz), applied to developing roach embryos from the moment of fertilization to gastrulation, decreased the length and weight of the yearlings [20]. The exposure of northern pike embryos to an SMF (10 mT) from fertilization, or the exposure of rainbow trout embryos to static (10 mT) and electromagnetic fields (1 mT, 50 Hz) from the eyed stage had no negative effect on their survival [12,17]. Similarly, no such effect was observed in the sea trout incubated in an SMF (5.5 mT) from fertilization, and the embryos’ survival was even higher [11]. Sinusoidal MFs (50 Hz) of 30 µT, 100 µT, 200 µT, 400 µT, and 800 µT had no effect on the cumulative mortality and malformation rate in zebrafish (*D. rerio*) embryos [21]. However, an MF of 500 Hz (1.4–1.6 µT) doubled the embryo mortality [24].

### 2.3. Permeability of Egg Envelopes

Mature teleost fish eggs are covered by a protective egg envelope, which, besides protection, plays an important part in gas exchange and ensures effective removal of metabolites [25]. Initially, the egg envelope is permeable, so that the egg placed in water, whether fertilized or not, absorbs water. As a result, perivitelline space is formed between the envelope and the egg cell (yolk cell sphere surrounded by cytoplasm with the haploid nucleus); it is filled with fluid that contains not only water but also cortical alveoli with hydrophile substances [25,26,27].

Osmometric measurements of egg envelopes of the sea trout, Atlantic salmon (*Salmo salar* L.), and rainbow trout in an SMF of 2 mT revealed changes in their permeability. The quantity of water passing through the envelopes placed on the osmometer collars in the SMF increased significantly; the field of this intensity caused an increase in permeability after egg activation during the formation of perivitelline space [26].

During such formation in sea trout, an STM of 250 mT slowed down water uptake through the envelope in the initial period (up to ca. 20 min) after egg activation. In the later period, there was a tendency to equalize the quantity of absorbed water, and the volume of eggs exposed in the static field was similar to that in the control 180 min after activation [27,28]. Sea trout eggs activated and exposed to SMFs (1 mT, 3 mT, 5 mT) for 24 h had a larger volume compared to the control. A similar increase in egg volume under the effect of an MF was observed in perch (*Perca fluviatilis* L.) and European whitefish. Eggs exposed in an MF in subsequent stages of embryogenesis did not differ from the control eggs [29].

### 2.4. Effect of MF on Canals in Egg Envelope (Zona Radiata)

In teleost eggs, the envelope (zona radiata) is built of two layers of different thickness, depending on the species: a thin external layer—the zona radiata externa (ZRE)—and a thicker internal layer—the zona radiata interna (ZRI) [25,30,31]. The external surface of zona radiata may bear pores—entrances to canals whose number and size are not always uniform over the whole surface [32]. The external layer of zona radiata in salmonid fishes is smooth and without visible structures; the internal layer is thicker and perforated by canals that are perpendicular to the surface. There is no distinct layered structure in the cross section, as opposed to other families and species [33]. The quantity of water penetrating through the egg envelope to the inside of the egg is probably dependent on changes in the number of open canals in the zona radiata externa (ZRE). The envelope in sea trout eggs, exposed in an MF of 5 mT within 2 h post-fertilization, had a greater number of open canals on the external surface of the zona radiata externa. The number of open canals on corresponding fragments of the zona radiata externa of the control eggs was 223 ± 18, and it was 283 ± 50 in the eggs exposed in the MF. During the subsequent 24 h post-fertilization, the number of open canals in the zona radiata externa at 3 mT and 5 mT decreased, while it was similar to the control at 1 mT [29].

### 2.5. Embryonic Motor Function

The movement of the yolk sphere with the developing embryo inside the egg plays a very important part in the normal course of embryonic development—it facilitates periblast aggregation and, first of all, enables the mixing of perivitelline fluid that favors gas exchange, thus improving the oxygen conditions of the developing embryo [34,35,36,37]. Embryonic motor activity appears at early stages of fish embryogenesis; it is manifest in different ways, depending on the species and stage of development. It starts with changes in the position of intracellular structures, followed by contractions of major systems of contractile elements; the contractions are manifest as grooves that pass in an undulating movement over the ectoplasm surface in early stages of embryonic development and are termed “quasi-peristaltic” (a quasi-peristaltic contraction refers to a movement performed by the egg cell ectoplasm; the movements are visible as a parallel translocation of crests on the yolk sphere surface. The translocations induce a swirling motion of the embryonic plate and revolving movements of the entire egg cell) [34,38,39,40,41]. This is followed by coordinated contractions of the spatially ordered specialized muscular layer of the soma and by movements in individual organs [35,36]. The first category of movements is based on periblast contractions. In northern pike eggs, it causes revolutions of the embryonic shield around the egg’s upper pole in the protoplast surrounding the yolk sphere, and a rotational movement that is the most distinct during gastrulation [34]. A similar phenomenon was observed in developing eggs of the medaka fish (*Oryzias latipes* Temminck & Schlegel). [35,36] In addition, in the embryonic development of the German carp (*Carasius auratus gibelio* (Bloch)), a protoplasmic wave occurs, and the duration of one cycle is about 2 min. [36]

#### 2.5.1. Ectoplasm Motor Function

**Figure 1:**
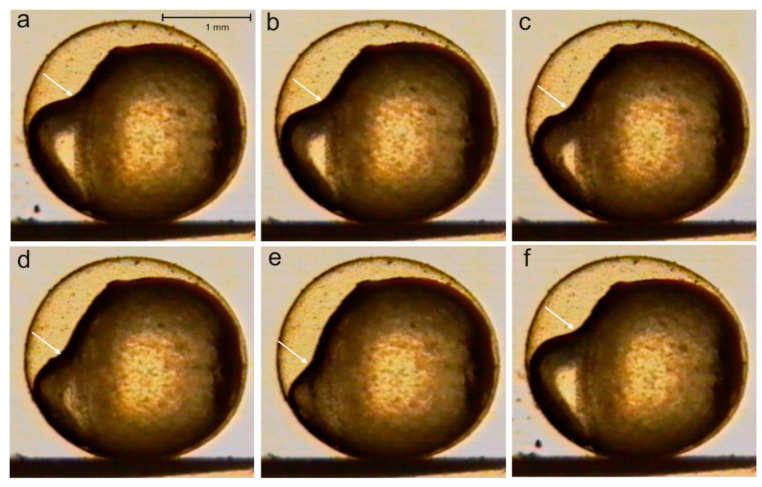
A quasi-peristaltic wave moving over the egg surface in the northern pike in a 4 mT static magnetic field (SMF); lateral view, ¾ epiboly; changes recorded at 1 min intervals indicated with an arrow (**a**–**f**); the moving wave causes changes in the embryo’s position inside the egg envelope [43].

Static or sinusoidal MFs have an effect on the quasi-peristaltic movements in the embryos of various fish families at the morula or gastrula stages, or at the stage of somatic movements [42,43]. The effect, however, is not always the same; there is a large body of data indicating that it is largely species-specific. In the northern pike, an SMF of 4 mT causes an increase in angular velocity of the peristaltic wave, and in early developmental stages, the effect is more pronounced (Figure 1) [42,43]. In the sea trout at the cleavage stage, no quasi-peristaltic wave was visible (control), and exposure in an SMF (4 mT) caused no noticeable changes. In embryos at the stage of ¼ epiboly, the MF in most cases caused a disappearance of the quasi-peristaltic wave [43]. A sinusoidal MF (15 mT, 50 Hz) caused either a complete disappearance of the quasi-peristaltic wave or (less often) an increase in the velocity of its cycle around the yolk sphere [43].

**Figure 2:**
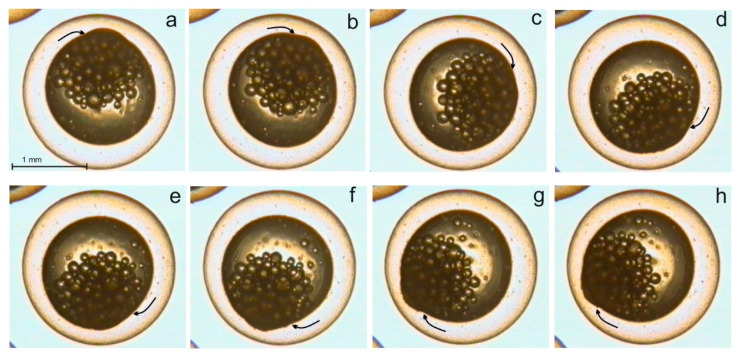
A quasiperistaltic wave moving at 1 min intervals (**a**–**h**) over the ectoplasm surface in the egg of European whitefish in a 4 mT SMF (top view, blastula stage) [43].

In some cases, a great regularity of the changes of quasi-peristaltic movements under the effect of SMF was observed. In the European whitefish in an SMF (4 mT), the angular velocity of a quasi-peristaltic wave in embryos at the blastula stage increased with each cycle. During the first six cycles (rotations), the increase was slow until achieving statistical significance during the sixth cycle (Figure 2). A similar tendency was observed at the gastrula stage—during the eighth cycle of the wave on the perimeter of the yolk sphere, the differences in angular velocity of the moving grooves and crests became statistically significant [43]. An EMF (15 mT, 50 Hz) caused an increase in the velocity of the quasi-peristaltic wave in the ectoplasm of the European whitefish, and significant differences occurred after the fifth cycle at the blastula stage, and after the seventh cycle at the gastrula stage. Moreover, the ectoplasm crests were observed to increase in height [43].

Similarly, in the big-scale sand smelt (*Atherina boyeri* Risso), exposure to an SMF (4 mT) increased the velocity of the quasi-peristaltic wave of the ectoplasm of embryos at the blastula stage, and the differences became statistically significant after the sixth cycle [43].

#### 2.5.2. Motor Function of Cardiac Muscle (Late Embryogenesis)

**Figure 3:**
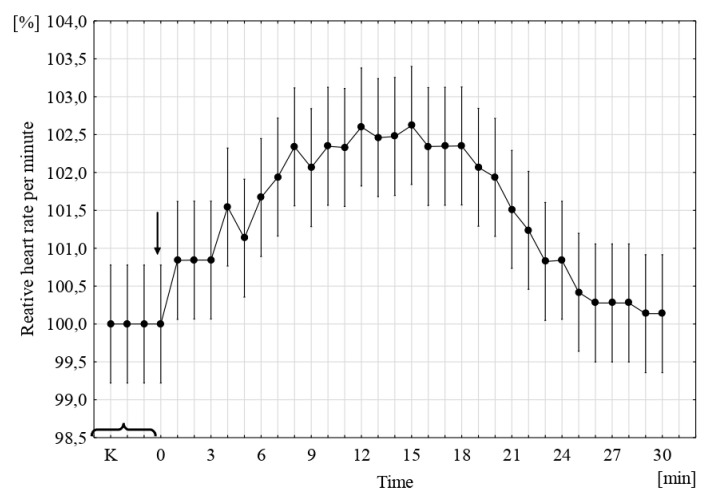
The relative number of cardiac contractions in sea trout embryos in the SMF (4 mT, *p* ˂ 0.001, vertical bars denote 95% CI); the number of contractions in the control was assumed to be 100%. K—control, 0—the point at which the MF was switched on [43].

SMFs and time-varying MFs have a different effect on the heart rate of fish embryos; it is to some extent species-specific. The exposure of sea trout embryos to an SMF (4 mT) initially (for ca. 10 min) caused an increase in the frequency of embryonic heart contractions; this was followed (during the next 10 min) by a leveling of the heartbeat and finally its decrease to the initial level (after 30 min), as in the control (Figure 3). European whitefish embryos in an SMF (4 mT) showed a slight and short-lasting slowing down of the heart rate, and the frequency then increased distinctly to reach its peak approximately from the 8th to the 10th minute; the acceleration was followed by a slowing down and stabilization at one level higher than the control [43]. The exposure of common carp (*Cyprinus carpio* L.) embryos to an SMF (51–70 mT) caused an increase in the heart rate already in the first minute. The fastest heart rate occurred within 5 min from exposure, and the frequency of contractions then decreased to reach the pre-experiment level after 15 min [44]. Northern pike embryos exposed to an SMF (4 mT) reacted similarly to common carp embryos: the heart reaction occurred immediately after the beginning of the exposure, followed by an increased heart rate during 5 min and then a slow decrease to the initial level [45]. The exposure of the big-scale sand smelt to an SMF (4 mT) evoked a reaction in the form of a slight increase in heart rate by ca. 2%, preceded by a temporary slow-down. This persisted for 3–4 min and was followed by a constant and regular decrease to the control or slightly lower level [43].

**Figure 4:**
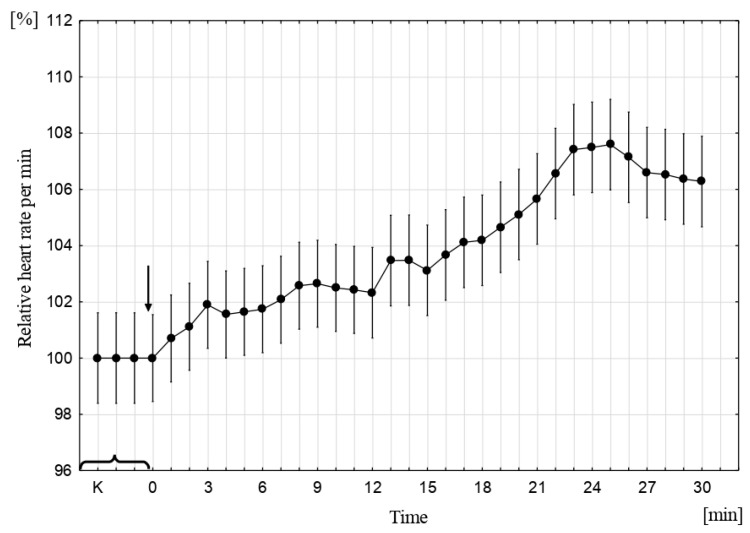
The relative number of cardiac contractions in sea trout embryos in alternating MF (15 mT, 50 Hz, *p* ˂ 0,001, vertical bars denote 95% CI); the number of contractions in the control was assumed to be 100%. K—control, 0—the point at which the MF was switched on [43].

In a time-varying MF (15 mT, 50 Hz), the reaction of the heart muscle in sea trout embryos was different: the embryo’s heart accelerated, and its contractions remained at a similar level from the third to the sixth minute, which was followed by a constant, steady increase until the 22nd minute; an increased heart rate was maintained until the end of the experiment (Figure 4).

In the European whitefish, as in the sea trout, the embryo’s heart in a time-varying MF (15 mT, 50 Hz, sinusoidal wave) accelerated, and a constant, steady increase was observed until the 22nd minute from the moment of exposure; the increased heart rate was maintained until the end of the experiment [43].

### 2.6. Embryonic Pigment Cells

Melanophores are star-shaped cells with numerous processes. Very important components of their structure are actin microfilaments and microtubules [46,47,48]. Melanosomes located in the melanophore cytoplasm are oval in shape and contain melanin, which protects the cell’s DNA against, e.g., ultraviolet radiation [49]. In embryonic development, in most cases, pigment cells become visible as early as ^2^/_3_ to ^3^/_4_ of the course of embryogenesis, and the quantity of pigment contained in them increases gradually. Initially, the greatest number of pigment cells occurs in the eyes, on the dorsal side of the embryo and in the yolk sac [50].

#### 2.6.1. Eye Pigment

**Figure 5:**
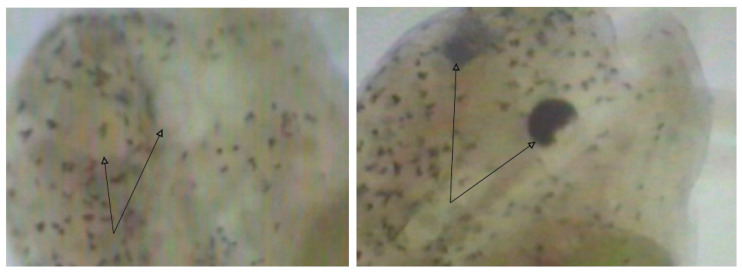
Eye pigmentation of African catfish larvae in (**left**) geomagnetic field (GMF) and (**right**) SMF—5 mT (used with kind permission from Adam Brysiewicz).

Constant exposure to an SMF from the moment of fertilization delayed the appearance of the pigment in European whitefish embryos, since melanophores in the eyes were visible from 92 D° in the embryos exposed in the fields of 1 and 3 mT and from 105 D° in those exposed in 5 mT, while in the control the first eye melanophores appeared after 86.5 D°. A similar delay in the appearance of eye melanophores was observed in the vendace: the first melanophores (in eyes) appeared in the control after 97.5 D°, and they appeared in an SMF of 1 mT after 11 D°, in 3 mT after 119.5 D°, and in 5 mT after 125 D° [51]. A similar dependence was observed in the African catfish (*Clarias gariepinus* Burchel)) (Figure 5).

#### 2.6.2. Body Pigment

MFs affect the time of appearance of pigment cells in the developing fish embryos, their number, and the degree of melanin concentration. The first body melanophores in the control group of European whitefish embryos appear on the surface of yolk sac and in the caudal region, and were observed after 129.5 D°. In the embryos exposed in an SMF from fertilization, the melanophores appeared later, and the moment of their appearance was correlated with the intensity of the field. With increasing intensity of the field, their appearance was increasingly delayed: they appeared at 146 D° in 1 mT, at 162.5 D° in 3 mT, and at 173.5 D° in 5 mT. Moreover, the MF caused a decrease in the number of pigment cells in the embryo’s body, and the value of the melanophore index (melanophore index: the value describing the degree of dispersion or aggregation of melanin inside pigment cells; 1: maximum aggregation; 5: maximum dispersion; 2, 3, and 4: intermediate stages) decreased with increasing intensity of the field [51]. A somewhat different reaction of pigment cells on the yolk sac and body was observed in developing vendace embryos: the first melanophores in the embryos incubated in an MF of 1 mT appeared after 158 D°. Body pigment appeared in the control embryos after 6 D° and in the embryos exposed in an MF of 3 mT after 172.5 D°. Melanophores in the embryos that developed in an MF of 5 mT were the last to appear (192 D°). Analysis of the degree of dispersion of melanin in pigment cells of vendace embryos exposed in SMFs of 1 mT, 3 mT, and 5 mT showed a tendency for pigment aggregation with increasing intensity of the field [51]. In sea trout embryos, SMFs of 1 mT, 3 mT, and 5 mT delayed the appearance of melanophores; the number of melanophores was also smaller. The first melanophores appeared after 184 D° and in the experimental variants after 194 D° (1 mT and 3 mT) or 198 D° (5 mT) [16]. The MF affects the time of appearance of pigment cells in the bodies of developing fish embryos, their number, and the degree of melanin concentration. In the black tetra (*Gymnocorymbus ternetzi* Boulenger, 1895), an MF of 14 T caused pigment aggregation in the cells, while a field of 8 T caused no such effect [52]. A time-varying MF (62 mT, 50 Hz) caused a delayed change of color in the goldfish aged less than 1 year after 20 h of exposure [53].

### 2.7. Respiration

An MF has an influence on respiratory processes in fish embryos, and this is correlated with the stage of embryogenesis at which embryonic motor function becomes apparent. Measurements of oxygen consumption in rainbow trout embryos in an SMF showed that the MF stimulated respiration, which was manifested as a significant increase in oxygen consumption [54,55]. In SMFs of 5 mT and 10 mT, oxygen consumption in rainbow trout embryos, compared to the control, increased during blastomerization, at the beginning of gastrulation, in the period after the closing of the blastopore, and during early organogenesis. In pre-hatching embryos, no statistically significant differences were found between the oxygen consumption in the MF and in the control [54]. In SMFs of 50 and 150 mT in rainbow trout, a distinct increase in oxygen consumption compared to the control occurred in the period of the eyed stage and the further development of the circulatory system and in fields of 150 and 300 mT during the closing of the blastopore. In a static field of 50 mT, differences in oxygen consumption were also observed in the period of blastomerization and at the beginning of gastrulation [25,26].

### 2.8. Directional Reactions of Embryos

The spatial orientation of embryos pertains not only to fish species that migrate for long distances, such as the Atlantic salmon and the sea trout, but also to species that perform no such migrations [56,57].

**Figure 6:**
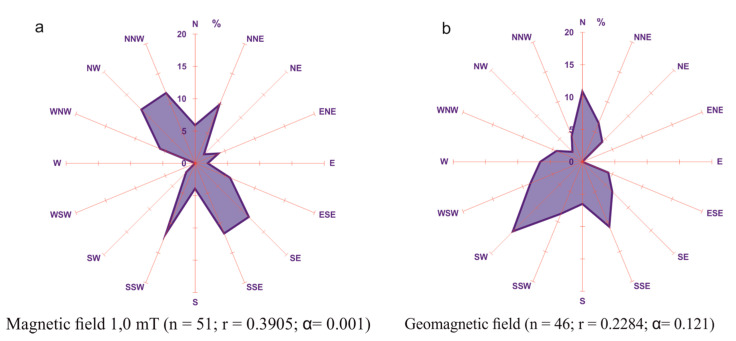
Spatial arrangement of northern pike embryos in an SMF of 1 mT (**a**) and a GMF (**b**) after 51 D°; the Reyleigh test [59].

Eggs of the Atlantic salmon, sea trout, rainbow trout, vendace, northern pike, and rudd (*Scardinus erythrophthalmus* L.) were incubated from the moment of fertilization to blastopore closing, in SMFs of 0.5, 1.0, 2.0, and 4.0 mT, superimposed on the local MF of the orientation in accordance with that of the GMF [58]. Studies have shown that the embryos of the migratory species (Atlantic salmon, sea trout, and rainbow trout) in the GMF and in the generated static fields of 0.5, 1.0, 2.0, and 4.0 mT superimposed on the GMF were always aligned mainly in north–south planes (N–S, NNE–SSW, and NNW–SSE) [58]. Likewise, embryos of the vendace and northern pike in MFs of 0.5, 1.0, and 4.0 mT as well as the rudd in MFs of 1.0, 2.0, and 4.0 mT superimposed on the GMF preferred the north–south alignment (Figure 6). No significant directional preferences were observed for embryos of the vendace and northern pike in a generated MF of 2.0 mT and of the rudd) in a field of 0.5 mT [59].

Exposure to SMFs, rotated clockwise by 90°, caused the embryos of Atlantic salmon, sea trout, rainbow trout, vendace, northern pike, and rudd to align according to the force lines of a generated magnetic field of 0.5 mT, which confirms the much stronger effect of the generated static field compared to the GMF [59]. The effect of exposure in static fields rotated by 90° in a later period of embryogenesis (not from the start of incubation) depended on the stage of embryogenesis. A magnetic field of higher intensity caused a more distinct orientation compared to the control. In the experimental variants, embryos of the sea trout and Atlantic salmon in a field of 2.0 mT, whose position was changed after 60 D° of development, aligned themselves along the north–south planes, while in the control variant, no significant orientation was observed [58,59].

Short-term (60 min) exposure of sea trout eggs prior to fertilization in a time-varying MF (100 mT, 50Hz, sinusoidal) and in an SMF (100 mT) had no effect on later directional preferences of the embryos [59].

## 3. Larvae

Static and time-varying MFs seem to have no effect on larval survival. Experiments on guppies (*Lebistes reticulatus* Peters) exposed to an MF of 50 mT for 200 days [60], northern pike exposed for 6 days after hatching to a static field (10 mT) [17], sea trout exposed after hatching to SMFs (1–5 mT) [11], and rainbow trout exposed from the eyed stage to the sixth day post-hatching to an SMF (10 mT) and an EMF (1 mT, 50 Hz) [12] showed that MFs of the values applied had no effect on their larval survival. An analysis of the mortality of flounder (*Platichthys flesus* (L.) fry in a magnetic field of 3.7 mT showed no significant differences between the experimental variants and the control [61]. Brewer [60], with continuous exposure of the guppy for 200 days and three consecutive generations, showed the effect of an MF of 50 mT on the reproduction rate in second and third generations.

The size and growth rate of fish larvae appear to be not or only slightly affected by MFs. Northern pike larvae exposed in an SMF of 10 mT were of similar size to the control variant. Immediately after hatching, their standard length in an experimental variant was 9.84 mm, and that in the control was 9.86 mm. The growth rate of the larvae six days after hatching was also similar: in the exposed larvae, it was 0.52 mm × day^−1^; in the control, it was 0.53 mm × day^−1^; the larval mortality was also similar: 54.5% for the experimental variant and 54.3% for the control [62]. The Fish Condition Index of the northern pike was smaller in an SMF 1 day after hatching, and the yolk sac height was smaller [17].

The exposure of eggs and larvae of the rainbow trout from the eyed stage to the 26th day after hatching in static (10 mT) and EMFs (1 mT, 50 Hz) caused no differences in larval growth between the exposed and control larvae. An enhanced yolk sac absorption rate was observed in both experimental variants; moreover, larvae with absorbed yolk sacs at the time of swimming were less efficient in food uptake [12].

A static field of 10 mT and a 50 Hz EMF of 1 mT caused developmental instability in the inner ear organ. The rainbow trout, incubated and reared in fields of such values for 13 days in the egg stage and 24 days in the larval stage, showed a fluctuating asymmetry of otolith size, and a higher significance occurred in the youngest larvae (five days after hatching) [63].

### 3.1. Heart Rate Studies

The exposure of common carp larvae in an SMF (51–70 mT) causes an increase in heart rate preceded by a temporary slow-down in the first minute of exposure. The fastest heart rate was observed within the first three minutes of the experiment, and it decreased to a level close to that before the experiment after seven minutes [11]. A similar change in heart rate in reaction to an SMF was observed in northern pike larvae—immediately after exposure, the heart rate slowed down and then accelerated, and this state persisted for 4 min, which was followed by a return to the original level [11].

### 3.2. Fin Motor Function

Larvae of many species, such as zebrafish, rainbow trout, and *Brycon amazonicus* (Spix et Agassiz), immediately after hatching, do not have fully formed respiratory organs in the form of gills. An additional respiratory function is fulfilled, among other organs, by pectoral fins [64]. Their motor function is to some degree correlated with the functioning of the circulation system, since a faster heart rate precedes increased fin mobility, thus increasing the quantity of oxygen, which reaches the larvae’s respiratory surfaces. When sea trout larvae were subject to an SMF (between 51 and 70 mT), a dependence was observed between the heart rate and the frequency of fin movement after the start of exposure. The heart rate was highest between the second and seventh minute of exposure, and the frequency of fin movements was highest between the third and ninth minute of the experiment [65].

### 3.3. Pigment Cells

In teleost larvae, the pigment cells play a very important role: they facilitate predator avoidance [65]. The number of pigment cells and the degree of dispersion of melanin in the melanophores change with development and under the effect of environmental factors [66]. The exposure of hatched northern pike larvae in an SMF influences the degree of aggregation or dispersion of melanin in their pigment cells, as expressed by the melanophore index. SMFs of 1 mT, 3 mT, and 5 mT, applied constantly irrespective of light intensity, caused statistically significant differences between a control and all experimental variants: the pigment aggregated in the melanophores [67]. A similar reaction occurred in skin pigment cells of European whitefish larvae—the degree of pigment dispersion in the melanophores was greatest in the control variant, and the value of the melanophore index decreased with the increasing intensity of MF (1 mT, 3 mT, and 5 mT) [51].

The incubation of river trout (*Salmo trutta* m. *fario* L.) larvae in an SMF of the same values but in light of greater intensity (75 lux) caused pigment aggregation, i.e., a smaller melanophore index, while no such aggregation was observed in less intense light (30 lux) [50]. In sea trout, the greatest pigment dispersion in the melanophores was observed in the control, and the smallest dispersion, i.e., the smallest melanophore index, was recorded in a field of 5 mT [16].

### 3.4. Directional Reactions of Larvae and Fry

Changes in the values of surrounding MFs may cause changes in directional reactions and orientation in the early development stages of fish. When embryos of the Chinook salmon (*Oncorhynchus tschawytscha* (Walbaum)) were placed in tubes imitating natural gravel nests, the emerging larvae were sensitive to an MF. Reversal of the vertical component of the field had a significant effect on their upward movement [68].

Studies on larvae of Atlantic haddock (*Melanogrammus aegrefinus* (Linnaeus)) placed in a behavioral chamber in the Norwegian North Sea and in a magnetic laboratory showed that, when the direction of MF was modified, the larvae aligned themselves in the northwest direction [69]. Sea trout larvae and fry in experimental sets where they could enter chambers with a modified value of MF at the entrance mostly entered chambers with an increased value of magnetic field [69]. Similar reactions were observed in the vimba bream (*Vimba vimba* (Linnaeus)) fry [70].

Observations of the behavior of juvenile Siberian sturgeon (*Acipenser baeri* Brandt) in round arenas where the fish could choose movement in four directions showed that magnetic fields of both 0.2 mT and 0.9 mT affected their directional reaction (in preparation).

Juvenile zebrafish, aged approximately 4 months, and roach showed a spatial orientation in a GMF. The zebrafish preferred north and south, and they preferred east and west after a 90° rotation of the horizontal component of the GMF. The roach in the geomagnetic preferred east-northeast only, and a 90° rotation of the horizontal component of the GMF changed the preferred direction to south-southeast [24].

The exposure of roach embryos in extremely LF-EMFs (1.4–1.6 µT, 500 Hz; 1.4–1.6 µT, 72.5 Hz) caused changes in the total number of vertebrate and the number of seismosensory system orifices in the mandibular bones [24].

## 4. Gametes

### Spermatozoa

There is a direct dependence between the spermatozoa motility and their ability to fertilize eggs, which is why sperm motility is one of the most important criteria in assessing fish semen quality. The movement capability of fish sperm after their release to the water is limited in time. The motility of fish spermatozoa is sensitive to several external factors, such as temperature, ions, pH, or osmolarity [71,72,73,74]. Salmonids are controlled by the concentration of K^+^ ions [75], *Acipenseridae* spermatozoa are immotile in the seminal plasma [76], and osmotic pressure is the main factor in cyprinids [77]. SMFs of 1 mT, 5 mT, and 10 mT and in time-varying MFs of the same intensity (50 Hz) were found to influence the sperm motility in sea trout; they affected curvilinear velocity (VCL), rectilinear velocity (VSL), and average velocity (VAP). In both static and time-varying fields, the exposure extended the time of sperm motility to 12 days (288 h), while the control sperm remained motile for only 3 days (72 h); in addition, the exposure increased the velocity [78].

An SMF improved sperm motility parameters in Danube salmon (*Hucho hucho* (L.))—among others parameters, VCL, VSL, and VAP. The percentage of the fertilization of eggs by spermatozoa kept for 24 h in an MF of 1 mT was 71.32%; it was 58.23% in an MF of 5 mT, –59.99% in an MF of 10 mT, and 32.60% in the control [78]. The effect of the exposure lasted during the hours following the sperm sampling [78,79]. The dispersion of genetic material (comet assay) in the heads of sea trout spermatozoa that had been subject to static and time-varying MFs was within the normal range, thus indicating no effect of the applied fields on DNA fragmentation [78].

## 5. Concluding Remarks

The interest in the effect of SMFs and EMFs on early developmental stages has been steadily increasing in recent years, since technological advancement contributes much magnetic and electromagnetic “pollution” in the aquatic environment, and the number of various electric facilities and industrial equipment in aquatic environment is growing. The effects of anthropogenic MFs on early development stages of fishes are varied and manifest both with a long-term and short-term exposure to an MF. The effect depends on the characteristics of the field (static vs. alternating), its magnitude, the time of exposure, and the advancement of ontogenesis during the exposure. The effect of long-term exposure to an SMF on, for example, the duration of the hatching period may be favorable because it reduces the duration of the process. Incubation in an SMF may increase survivorship of the hatchlings. Storing sperm in an MF prolongs its activation capacity. An alternating MF, depending on its characteristics, can increase the embryos’ mortality and can cause a heart rate disturbance or a developmental instability of the inner ear organ. The long-term impact of anthropogenic SMFs and EMFs on early developmental stages, which in consequence affects whole fish populations, should be considered, as they even offer the possibility of estimating the effect on whole fish populations. There is thus a need for standards for SMFs and EMFs that can be safely introduced into aquatic environments.

## Data Availability

There is not Data Availability Statements.
